# Relative Efficacy of Different Exercises for Pain, Function, Performance and Quality of Life in Knee and Hip Osteoarthritis: Systematic Review and Network Meta-Analysis

**DOI:** 10.1007/s40279-019-01082-0

**Published:** 2019-03-04

**Authors:** Siew-Li Goh, Monica S. M. Persson, Joanne Stocks, Yunfei Hou, Nicky J. Welton, Jianhao Lin, Michelle C. Hall, Michael Doherty, Weiya Zhang

**Affiliations:** 1 Arthritis Research UK Pain Centre, Academic Rheumatology, University of Nottingham, Clinical Sciences Building, City Hospital, Nottingham, NG5 1PB UK; 2 Sports Medicine Unit, University of Malaya, Kuala Lumpur, Malaysia; 3 Department of Orthopaedic Surgery, Peking University People’s Hospital, Beijing, China; 4 Population Health Sciences, University of Bristol, Bristol, UK; 5 Division of Physiotherapy Rehabilitation Sciences Education, University of Nottingham, Nottingham, UK

## Abstract

**Background:**

Guidelines recommend exercise as a core treatment for osteoarthritis (OA). However, it is unclear which type of exercise is most effective, leading to inconsistency between different recommendations.

**Objectives:**

The aim of this systematic review and network meta-analysis was to investigate the relative efficacy of different exercises (aerobic, mind–body, strengthening, flexibility/skill, or mixed) for improving pain, function, performance and quality of life (QoL) for knee and hip OA at, or nearest to, 8 weeks.

**Methods:**

We searched nine electronic databases up until December 2017 for randomised controlled trials that compared exercise with usual care or with another exercise type. Bayesian network meta-analysis was used to estimate the relative effect size (ES) and corresponding 95% credibility interval (CrI) (PROSPERO registration: CRD42016033865).

**Findings:**

We identified and analysed 103 trials (9134 participants). Aerobic exercise was most beneficial for pain (ES 1.11; 95% CrI 0.69, 1.54) and performance (1.05; 0.63, 1.48). Mind–body exercise, which had pain benefit equivalent to that of aerobic exercise (1.11; 0.63, 1.59), was the best for function (0.81; 0.27, 1.36). Strengthening and flexibility/skill exercises improved multiple outcomes at a moderate level. Mixed exercise was the least effective for all outcomes and had significantly less pain relief than aerobic and mind–body exercises. The trend was significant for pain (*p* = 0.01), but not for function (*p* = 0.07), performance (*p* = 0.06) or QoL (*p* = 0.65).

**Conclusion:**

The effect of exercise varies according to the type of exercise and target outcome. Aerobic or mind–body exercise may be the best for pain and function improvements. Strengthening and flexibility/skill exercises may be used for multiple outcomes. Mixed exercise is the least effective and the reason for this merits further investigation.

**Electronic supplementary material:**

The online version of this article (10.1007/s40279-019-01082-0) contains supplementary material, which is available to authorized users.

## Key Points

**Table:** 

The effect of exercise in knee and hip osteoarthritis depends on type of exercise and outcome of interest.
Aerobic and mind–body exercises appear to be the two most effective exercise therapies for pain and function, whereas strengthening and flexibility exercises appear to be good for moderate improvement of multiple outcomes.
Mixed exercise is the least effective exercise. However, it may be used for patients who do not respond to other types of exercise therapy because it is still better than no exercise control for all four patient-centred outcomes.

## Introduction

Pain from knee and hip osteoarthritis (OA) can have a significant impact on the physical function and quality of life (QoL) of affected individuals worldwide [1]. Exercise is one of the core therapies for OA [2] to improve pain and function [3, 4]. Existing evidence indicates that the magnitude of response varies according to the type of exercise (e.g. strengthening, aerobic etc.) [5]. However, little is known about the relative efficacy between different exercises for different outcomes.

Most randomised controlled trials (RCTs) compare exercise regimens against non-exercise interventions, and direct comparisons between different exercises are uncommon. This is because a head-to-head comparison trial is very costly and it is impractical to undertake RCTs to examine the relative effects between all types of exercises. Alternatively, network meta-analysis (NMA) can indirectly compare multiple interventions through a common comparator when head-to-head RCTs are sparse or absent [6]. It utilises all available evidence in the network, both direct and indirect, to enhance the power of the estimation [7].

Previously, Uthman et al. [8] undertook a sequential analysis and NMA to examine whether there was sufficient evidence to support the use of exercise for people with lower limb OA, and whether one exercise was better than another. They found that up to 2002, sufficient evidence existed to show a significant benefit of exercise over no exercise. Strengthening exercise yielded the largest effect size for pain outcomes, whereas a combined intervention of strengthening, flexibility and aerobic exercise had the largest effect size for function. However, no performance or QoL measures were included.

In this review, we aimed to extend the work of Uthman et al. [8] by updating the evidence, expanding the outcomes to include objective performance measures and QoL, and refining the exercise classification to include mind–body exercise such as tai chi and yoga.

## Methods

### Search Strategy and Selection Criteria

This NMA is part of a larger review that included RCTs comparing all forms of exercise to non-exercise interventions, or to another exercise type. Detailed inclusion criteria for the larger review are available in our registered and published protocol (PROSPERO CRD42016033865) [9]. The specific inclusion criteria for this NMA were RCTs that (1) recruited participants with knee OA, hip OA, or mixed knee and hip OA diagnosed clinically and/or radiographically; (2) assigned exercise programmes without additional active treatment (e.g. analgesics) as the intervention; (3) assigned usual care/waiting list or a different exercise as the control group; and (4) measured at least one outcome for pain, function, objective performance or QoL.

The systematic search was conducted in December 2015 and updated in December 2017. Nine electronic databases (Allied and Complementary Medicine Database (AMED), Cochrane Central Register of Controlled Trials (CENTRAL), Cumulative Index to Nursing and Allied Health Literature (CINAHL), Excerpta Medica Database (EMBASE), MEDLINE Ovid, Physiotherapy Evidence Database (PEDro), PubMed, SPORTDiscus and Google Scholar) were searched for peer-reviewed publications without language or publication date limitations. As an example, the Medline search strategy is shown in Electronic Supplementary Material (ESM) Appendix 1. The reference lists of systematic review protocols published in Cochrane Library since 2014 were used to supplement the electronic database search. Publication of study protocols were flagged pending the full publication of the trials.

Selection of relevant studies and subsequent data extraction was undertaken by a single reviewer (SLG), with advice from a second reviewer (MH) should queries arise. A third reviewer (WZ) was involved if agreement could not be reached. Data extraction was compared between SLG and either MSMP, JS or YFH in a random sample (10%) of selected studies. Should disagreement be over 5% of the total extracted variables, the whole set of the studies would be double extracted, otherwise the single extraction was used; that is, a maximum 5% disagreement was allowed for data extraction.

### Interventions

Exercises were classified into muscle strengthening, aerobic, or flexibility/neuro-motor skills training (flexibility/skill) according to the American College of Sports Medicine (ACSM) recommendation [10]. Strengthening exercises are exercises that aim to increase force of muscle contraction (e.g. lifting dumbbells, squats); aerobic exercises to improve cardiorespiratory endurance (e.g. swimming, jogging); flexibility exercises to improve joint range of motion and muscle pliability (e.g. hamstring stretch, gastrocnemius stretch); and neuromotor skills training to improve balance and coordination (e.g. wobble board, walking on foam). In addition, an exercise programme was classified as mind–body exercise if it integrated mindfulness/relaxation into physical movements (e.g. tai chi, yoga), and classified as mixed exercise when it included more than one core exercise type mentioned above, or when the authors did not specify it as a single component exercise.

‘Usual care’ control was determined based on the report. In ‘usual care’, participants were expected to continue the routine standard of care provided by their general practitioners. Control groups that were not given any specific intervention such as ‘waiting list’ or usual physical activity or where the authors did not specify the nature of the control were also classified as ‘usual care’. ‘Waiting-list’ controls were given active intervention after a period of observation, with no new intervention being delivered during the trial period.

### Outcomes

Our primary outcome of interest was pain, and secondary outcomes were self-reported function, objective performance (e.g. walking speed, strength, range of motion), and QoL. The primary time point was 8 weeks after commencement of the exercise regimen or the time point nearest to this. Eight weeks was chosen because it was the most frequently reported time point. When more than one scale was presented for pain, function or QoL, the more comprehensively reported scale was selected in the ranking order proposed by Fransen and McConnell [4] and Regnaux et al. [11].

For the performance, gait and walking parameters (e.g. walking distance, walking time, etc.) were prioritised. This was because the measurement and reporting of these parameters were relatively standard across trials compared with other performance outcomes such as strength or power. Limb-specific parameters, such as strength, power, or range of motion were only used if gait parameters were not available. Strength parameters extracted were, in descending order of preference, knee extensors, knee flexors, hip abductors, and then other muscle groups. When tests performed at varying intensities were reported, the results from the highest intensity tests were chosen.

### Data Analysis

The standardised mean difference of the change score (end-point minus baseline score) was used to estimate the effect size (ES). Standard deviations (SD) were imputed for trials that did not provide the SD or did not provide sufficient information to calculate the SD. The missing SD was imputed using the largest SD of the same scale reported in other trials if available, otherwise an arithmetic mean of other SDs was used [12].

A Bayesian random effects NMA model for continuous outcome data was used for the primary analysis. The WinBUGS codes were adapted from Dias et al. [13] and are provided in ESM Appendix 2. The posterior mean of the ES was reported with its 95% credibility intervals (CrI). Bayesian NMA produces simulations that allow interventions to be ranked from first to sixth. The median ranking and corresponding 95% CrI was generated alongside the pooled ES to identify the most effective exercise choice [14]. The significance of the ES hierarchical trend was assessed using meta-regression analysis [15].

Non-informative prior distributions were used and three Markov chains were run simultaneously. The initial 40,000 simulations were discarded as the burn-in period and the subsequent 120,000 simulations were used. Inspection of Gelman–Rubin tracing was performed to ensure that convergence or stabilisation of the simulations had been achieved.

Model fit, a measure of how well predictions from the model were supported by the observed data, was assessed. Consistency in the network was assessed by the node-splitting method [16] and design by treatment forest plot [17] based on frequentist analysis. The node-splitting method examines the agreement between direct and indirect comparisons. Design by treatment forest plot, on the other hand, visually demonstrates agreement between studies of different designs (e.g. whether estimation between A and C, obtained from two-arm design, is consistent with those obtained from multi-arm ABC or ACD designs). Data were processed and analysed using Microsoft Access, Excel, Stata (StataCorp. 2017. Stata Statistical Software: Release 15. College Station, TX, USA: StataCorp LLC), and WinBUGS (Version 1.4.3).

### Sensitivity Analysis and Subgroup Analysis

A modified Cochrane risk of bias assessment tool was used to assess study quality. Sensitivity analyses were performed on two of the items with the highest risk of bias and also on studies for which SD had been imputed. Subgroup analyses were performed to assess the efficacy at different joints (knee OA versus hip OA) and for different patient contexts, such as participants awaiting total joint replacement (TJR) versus participants not awaiting TJR.

## Results

**Fig. 1:**
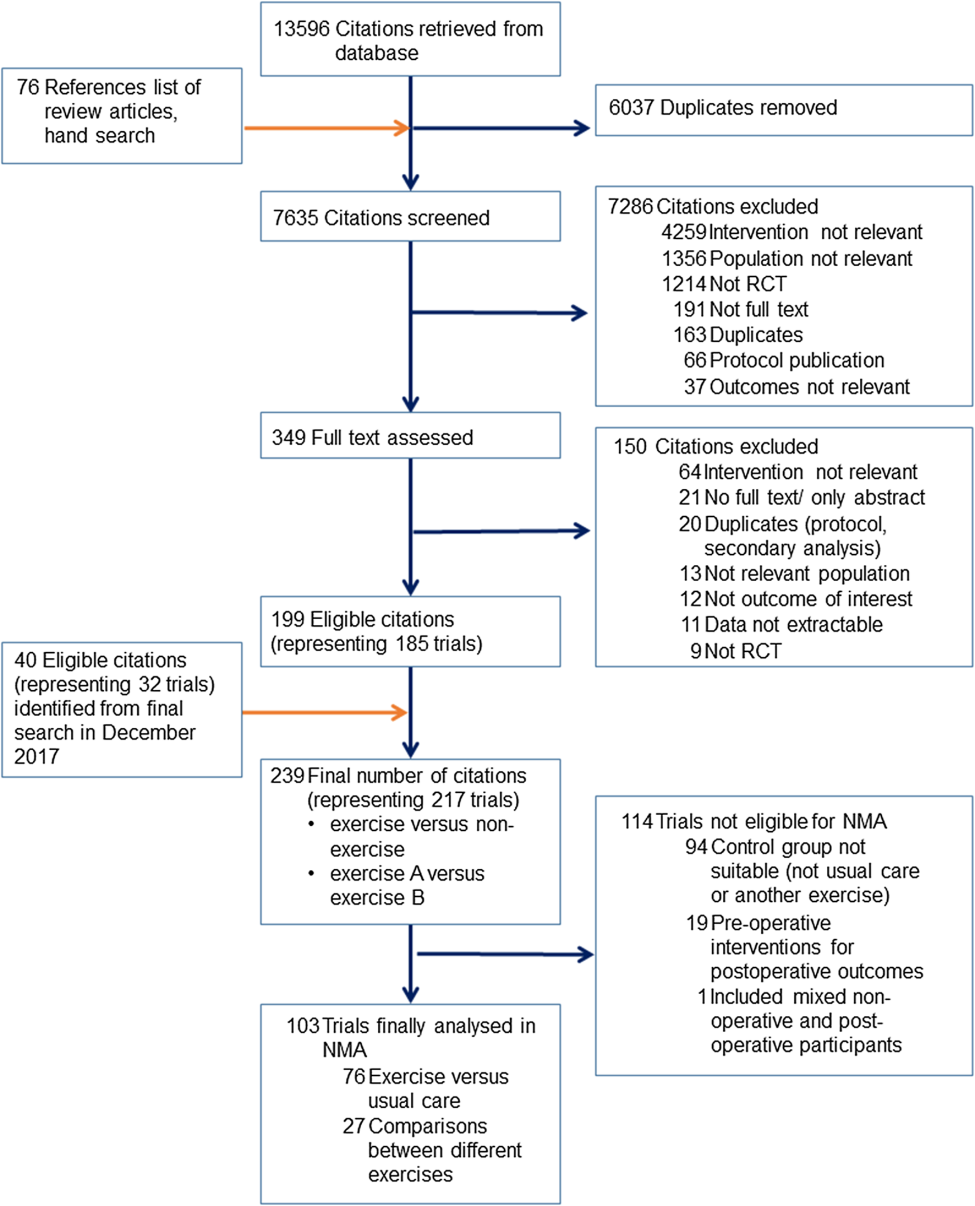
Study flow diagram for comparison between exercise and usual care and between different exercises. *NMA* network meta-analysis, *RCT* randomised controlled trials

**Fig. 2:**
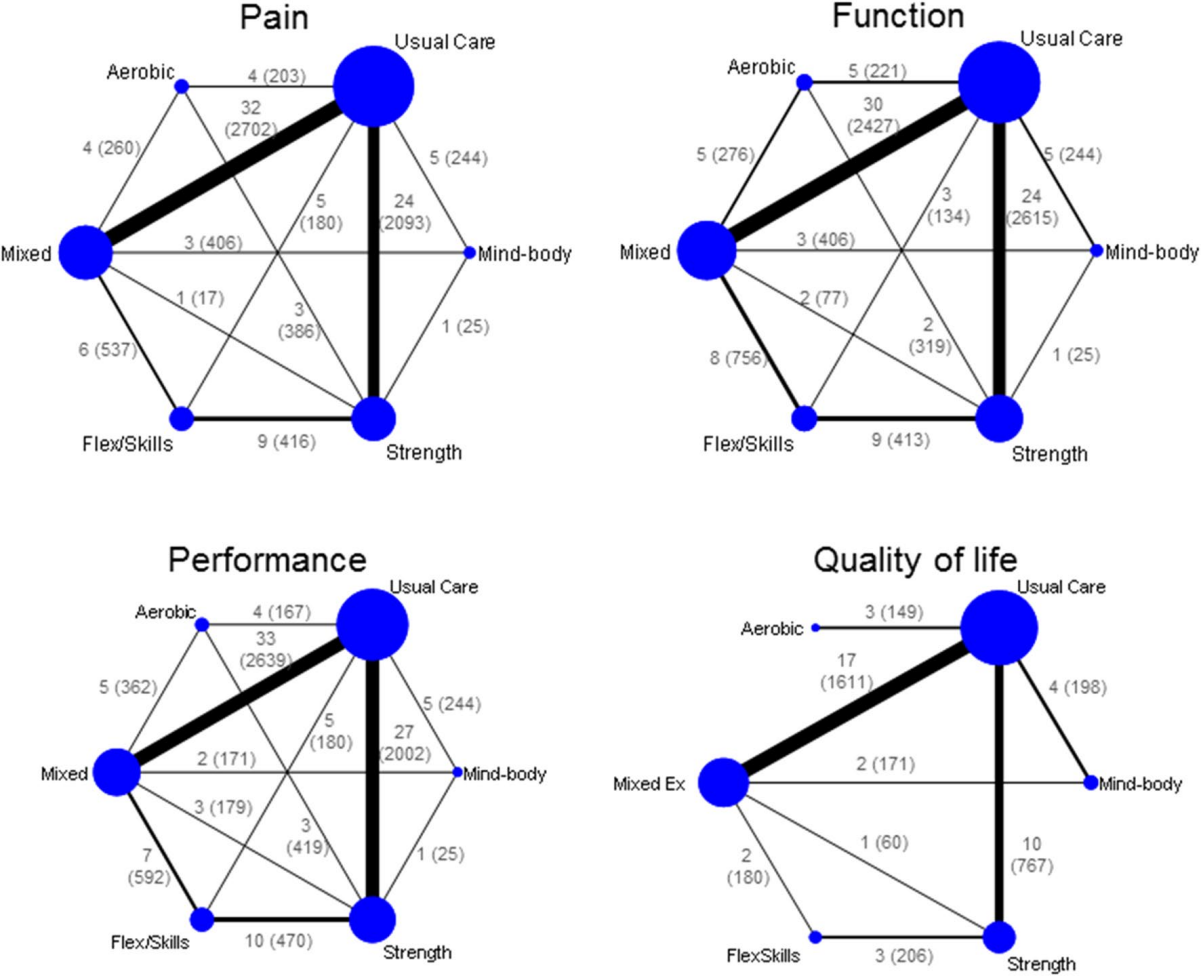
Network of direct comparisons formed by included studies. The size of nodes and lines connecting the nodes are proportionate to the number of participants and the number of trials, respectively. Data represent number of trials (number of participants). *Flex/Skills* flexibility and skills or neuromuscular training

From the initial 13,596 citations retrieved from the databases and 76 hand searches, we identified 239 articles (217 trials) to be eligible under the broader search strategy that included all types of non-exercise comparators including other non-pharmacological therapies or drugs (Fig. 1). Since the present NMA only considered trials comparing the five defined exercises with usual care or each other, only 103 trials (9134 participants) were included [18–130]. Of these, 76 (74%) trials used usual care as the control and 27 were head-to-head comparisons. Disagreement for double extraction of data was within the acceptable limit, so predominantly single extraction was retained. The characteristics of the included trials are listed in Table 1. Pain was assessed in 89 trials (7184 participants), function in 87 trials (7153 participants), performance in 95 trials (6760 participants), and QoL in 40 trials (3190 participants) (Table 2). Preliminary assessment of funnel plots identified one outlying study for pain [112] and another for QoL [48]. Both studies showed strong positive effects (ES > 5), very different from other studies. These studies were subsequently excluded from the main analysis. Egger’s statistical test is suggestive of publication bias (*p* < 0.05) for all outcomes except QoL (ESM Appendix 3). Figure 2 demonstrates the network for pain, function, performance and QoL. The comparisons were most seen between strengthening versus usual care, as well as between mixed exercise versus usual care.

**Table 1:** Characteristics of included studies

Study	Age (SD)	BMI (SD)	*n* (fem %)	Jt	Diagnosis	Pre-surgical	Study arms			Tool and outcome time point (month)			
							1	2	3	Pain	Function	Performance	QoL
Abbott et al. [18]	67 (10)	29 (6)	206 (55)	K, H	A	N	MEx	UC	–	Pain intensity score (0–10), 12	WOMAC (0–240), 2.25	40-m walk time (s), 12	–
Aglamis et al. [19]^a^ Aglamis et al. [20]	56 (5)	33 (5)	31 (100)	K	A, I	N	MEx	UC	–	VAS (0–10), 1.5	WOMAC (total), 1.5	6MWT (m), 1.5	SF-36 (0–100) general health (0–100), 1.5
An et al. [21]	65 (7)	26 (3)	28 (100)	K	A	N	MB	UC	–	WOMAC pain, 2	WOMAC (physical fx), 2	6MWT (m), 2	SF-36 mental health (0–100), 2
Aoki et al. [22]	73 (6)	26 (3)	36 (100)	K	I	Y	Fl/Sk	UC	–	VAS (0–100), 3	–	Walking speed (m/min), 3	–
Arnold and Faulkner [23]	74 (6)	30 (5)	83 (68)	H	A	N	MEx	UC	–	Arthritis Impact measurement scale –2, results not reported	Activity specific balance confidence (0–100), 2.75	6MWT (m), 2.75	–
Beaupre et al. [24]	67 (7)	32 (6)	131 (55)	K	UClr	Y	Str	UC	–	WOMAC pain (0–100), 1.5	WOMAC fx (0–100), 1.5	Quadriceps strength, 1.5	SF-36 mental health, 1.5
Bennell et al. [25]	65 (8)	28 (4)	89 (48)	K	A, I	N	Str	UC	–	NRS (0–10), 3.25	WOMAC fx (0–68), 3.25	Timed stair task (s), 3.25	–
Bennell et al. [26]	62 (7)	30 (4)	100 (52)	K	I	N	Fl/Sk	Str	–	VAS (0–100), 3	WOMAC fx (0–68), 3	Walking speed (m/s), 3	AQoL (− 0.04–1), 3
Bieler et al. [27]	70 (–)	27 (–)	152 (68)	H	A	N	Ae	MEx	Str	WOMAC pain, not extractable	WOMAC fx, not extractable	6MWT (m), 2	SF-36, not extractable
Börjesson et al. [28]	64 (5)	– (–)	68 (50)	K	I	Y	MEx	UC	–	NRS (0–10), 3	–	Walking speed (m/s), 3	–
Braghin et al. [29]	60 (–)	31 (–)	42 (74)	K	I	N	MEx	UC	–	WOMAC pain, 2	WOMAC fx, 2	Timed movement (s), 2	–
Bruce-Brand et al. [30]	64 (5)	33 (4)	26 (42)	K	I	Y	Str	UC	–	WOMAC pain (0–20), 1.5	WOMAC fx, 1.5	Timed walk (s), 1.5	SF-36 MCS, 1.5
Calatayud et al. [31]	50 (–)	32 (–)	50 (–)	K	A, I	Y	Str	UC	–	VAS (0–10), 2	WOMAC fx, 2	Timed up and go (s), 2	SF-36 physical fx, 2
Chaipinyo and Karoonsupcharoen [32]	66 (7)	25 (4)	48 (77)	K	A	N	Fl/Sk	Str	–	KOOS pain (0–100), 1	KOOS fx daily activity (0–100), 1	Timed walk (s), 1	KOOS QoL (0–100), 1
Chen et al. [33]	63 (7)	– (–)	120 (85)	K	A, I	N	Str	UC	–	VAS (0–10), 2	Lequesne’s index (1–26), 2	Knee range of motion, 2	–
Cheung et al. [34]	72 (8)	29 (7)	83 (84)	K	A	N	MB	MEx	–	VAS (0–10), 2	WOMAC fx (0–68), 2	Walking 50 ft (s), 2	SF-12 MCS, 2
Chopp-Hurley et al. [35]	54 (–)	29 (–)	24 (79)	K, H	A	N	Str	UC	–	ICOAP, not extractable	HOOS/KOOS, not extractable	6MWT (m), 3	HOOS/KOOS, not extractable
Christensen et al. [36]^a^ Henriksen et al. [37]	63 (6)	37 (–)	192 (81)	K	I	N	MEx	UC	–	VAS (0–100), 12	KOOS (daily fx) (0–100), 12	6MWT (m), 12	SF-36 mental health (0–100), 12
Cochrane et al. [38]	70 (7)	30 (–)	312 (63)	K, H	UClr	N	MEx	UC	–	WOMAC pain (0–96), 6	WOMAC fx (0–96), 6	Timed walk (s), 12	SF-36 mental health (0–100), 6
Cheung et al. [39]	72 (6)	29 (–)	36 (100)	K	A	N	MB	UC	–	WOMAC pain (0–20), 2	WOMAC fx (0–68), 2	8-min walk (m), 2	SF-12 MCS (0–100), 2
D’Lima et al. [40]	70 (6)	– (–)	30 (47)	K	UClr	Y	Ae	Mex	UC	–	Hospital for Special Surgery Knee rating (0–100), 1.5	–	–
Duracoglu et al. [41]	– (–)	– (–)	66 (100)	K	A, I	N	MEx	Str	–	–	WOMAC fx, 2	Timed walk (s), 2	SF-36 vitality, 2
Ebnezar et al. [42]^a^ Ebnezar and Yogitha [43] Ebnezar et al. [44] Ebnezar et al. [45]	60 (10)	– (–)	250 (70)	K	A, I	N	MB	MEx	–	NRS (0–10), 3-month	WOMAC (0–96), 3	–	–
Espejo Antunez et al. [46]	84 (8)	– (–)	31 (77)	K	A	N	MEx	UC	–	VAS, 1-month	WOMAC fx (0–96), 1	–	SF-36 mental health, 1
Ettinger et al. [47]	69 (6)	– (–)	439 (70)	K	I	N	Ae	Str	–	Likert scale (1–6), 3	Self-reported physical disability (1–5), 3	6MWT (m), 3	–
Evcik and Sonel [48]	56 (6)	– (–)	90 (62)	K	I	N	Ae	UC	–	VAS (0–100), 6	WOMAC fx (0–68), 6	–	NHP—emotional reaction, 6, outlier
Evgeniadis et al. [49]	68 (4)	34 (–)	53 (79)	K	A, I	Y	Str	UC	–	SF-36 body pain, 1	SF-36 physical fx, 1	Active range of motion (flexion), 1	SF-36 mental health, 1
Ferrara et al. [50]	63 (8)	– (–)	23 (61)	H	UClr	Y	MEx	UC	–	VAS (0–10), 1	WOMAC fx (0–68), 1	Quads strength, 1	SF-36 mental health, 1
Fitzgerald et al. [51]	64 (9)	30 (–)	183 (67)	K	A, I	N	Fl/Sk	MEx	–	NRS (0–10), 2	WOMAC fx (0–68), 2	Up and go (s), 2	Global rating of change, 2
Fransen et al. [52]	67 (9)	29 (5)	126 (73)	K	I	N	MEx	UC	–	WOMAC pain (0–100), 2	WOMAC fx (0–100), 2	Gait speed (cm/s), 2	SF-36 mental health, 2
Fransen et al. [53]	70 (6)	30 (–)	152 (74)	K, H	A	N	MB	MEx	UC	WOMAC pain (0–100), 3	WOMAC fx (0–100), 3	Timed walk (s), 3	SF-12 MCS (mean ± SD 50 ± 10), 3
French et al. [54]	62 (10)	– (–)	131 (64)	H	A, I	N	MEx	UC	–	NRS, 2	WOMAC fx (0–68), 2	50-ft walk test (s), 2	SF-36 mental health (0–100), 2
Ghroubi et al. [55]	41 (10)	38 (–)	56 (–)	K	I	N	MEx	UC	–	VAS pain (0–10), 2	WOMAC, 2	6MWT (m), 2	–
Gomiero et al. [56]	62 (–)	24 (–)	64 (95)	K	A	N	Fl/Sk	Str	–	VAS (0–100), 4	WOMAC (total), 4	Timed up and go (s), 4	SF-36 mental health, 4
Gondhalekar and Deo [57]	63 (6)	– (–)	30 (50)	K	A, I	N	Fl/Sk	Str	–	VAS (0–10), 0.75	WOMAC, 0.75	Hip extensor strength, 0.75	–
Gur et al. [58]	56 (12)	– (–)	23 (–)	K	I	N	Str	UC	–	NRS (0–10), 2	NRS of functional capacity (0–10), 2	15-m walk (s), 2	–
Hasegawa et al. [59]	77 (4)	24 (4)	28 (64)	K	UClr	N	MEx	UC	–	NRS (0–10), 3	–	Time up and go (s), 3	–
Henriksen et al. [60]^a^ Henriksen et al. [61]	64 (8)	29 (–)	60 (80)	K	I	N	MEx	UC	–	KOOS pain (0–100), 3	KOOS fx (0–100), 3	Walking speed (m/s), 3	KOOS QoL (0–100), 3
Hermann et al. [62]	70 (8)	28 (5)	80 (65)	H	UClr	Y	Str	UC	–	HOOS pain, 2.5	HOOS ADL, 2.5	Quadriceps power, 2.5	HOOS QoL, 2.5
Hinman et al. [63]	62 (9)	33 (7)	71 (68)	K, H	A, I	N	MEx	UC	–	VAS (0–10) on movement, 1.5	WOMAC fx (0–1700), 1.5	6MWT (m), 1.5	Assessment of QoL (− 0.04 to 1), 1.5
Hiyama et al. [64]	73 (5)	24 (–)	40 (100)	K	UClr	N	Ae	MEx	–	Japanese Knee Osteoarthritis Measure pain (0–125), 1	Japanese Knee Osteoarthritis Measure (total) (0–125), 1	Timed up and go (s), 1	–
Hoogeboom et al. [65]	76 (4)	27 (–)	21 (67)	H	UClr	Y	MEx	UC	–	VAS, 1	HOOS ADL fx (0–100), 1	6MWT (m), 1	HOOS QoL (0–100), 1
Huang et al. [66]	62 (5)	– (–)	132 (71)	K	A, I	N	MEx	UC	–	VAS (0–10), 2	Lequesne index, 2	Walking speed (m/s), 2	–
Huang et al. [67]	65 (6)	– (–)	140 (81)	K	A	N	Str	UC	–	VAS (standing/walking) (0–10), 2	Lequesne index (1–26), 2	Walking speed (m/min), 2	–
Huang et al. [68]	68 (6)	25 (2)	250 (80)	K	A	N	Str	UC	–	VAS (0–100), 3	WOMAC (0–68), 3	–	–
Hunt et al. [69]	66 (11)	27 (–)	17 (53)	K	A, I	N	Str	UC	–	–	–	Walking speed (m/s), 2.5	–
Ikuta et al. [70]	72 (6)	– (–)	27 (100)	K	UClr	N	Fl/Sk	MEx	–	KOOS pain, 1	KOOS ADL, 1	Walking speed (km/h), 1	SF-36 mental health, 1
Jan et al. [71]	63 (7)	– (–)	98 (81)	K	A, I	N	Str	UC	–	WOMAC pain (0–20), 2	WOMAC fx (0–68), 2	Timed walk (s), 2	–
Jan et al. [72]	63 (7)	– (–)	106 (69)	K	A, I	N	Str	UC	–	–	WOMAC fx (0–68), 2	Timed walk (s), 2	–
Jorge et al. [73]	61 (7)	31 (4)	60 (100)	K	A	N	Str	UC	–	VAS (0–10), 1.5	WOMAC (0–68), 1.5	6MWT (m), 1.5	SF-36 mental health (0–100), 1.5
Juhakoski et al. [74]	67 (6)	– (–)	118 (70)	H	A, I	N	MEx	UC	–	WOMAC pain (0–100), 3	WOMAC fx (0–100), 3	6MWT (m), 3	SF-36, not reported
Koli et al. [130]	59 (4)	27 (4)	78 (100)	K	I	N	Ae	UC	–	KOOS pain (0–100), 12	KOOS fx (0–100), 12	Strength, 12	KOOS QOL (0–100), 12
Krasilshchikov et al. [75]	58 (5)	28 (5)	16 (100)	K	I	N	MEx	UC	–	WOMAC pain, 2	WOMAC fx, 2	6MWT (m), 2	–
Krauss et al. [76]^a^ Steinhilber et al. [77]	59 (10)	27 (4)	218 (40)	H	A	N	MEx	UC	–	WOMAC pain (0–100), 3	WOMAC fx (0–100), 3	Hip and peak torque, 3	SF-36 mental health (0–100), 3
Kreindler et al. [78]	– (–)	– (–)	32 (75)	K	UClr	N	Str	UC	–	–	–	Quadriceps strength (180), 1.5	–
Kumar et al. [79]	53 (6)	25 (3)	44 (57)	K	UClr	N	Fl/Sk	Str	–	NRS, 1	WOMAC fx, 1	Joint position sense error, 1	–
Lee and Lee [80]	76 (6)	– (–)	46 (78)	K	UClr	N	MB	UC	–	WOMAC pain (0–96), 3	WOMAC fx (0–96), 3	Timed up and go test (s), 3	–
Lee et al. [81]	69 (5)	– (–)	44 (93)	K	I	N	MB	UC	–	WOMAC pain (26–130), 2	WOMAC fx (0–100), 2	6MWT (m), 2	SF-36 mental health (0–100), 2
Lim et al. [82]^b^ (neutral, malaligned)	66 (8)	29 (–)	107 (55)	K	A, I	N	Str	UC	–	WOMAC pain (0–100), 3	WOMAC fx (0–100), 3	Stair climb (s), 3	–
Lin et al. [83]	62 (7)	– (–)	89 (70)	K	I	N	Fl/Sk	Str	–	–	WOMAC fx (0–69), data not extractable	Knee extensor torque (180^o^), 2	–
Lin et al. [84]	63 (7)	– (–)	108 (69)	K	I	N	Fl/Sk	Str	UC	WOMAC pain (0–20), 2	WOMAC fx (0–68), 2	Timed walk (s), 2	–
Lund et al. [85]	68 (14)	– (–)	79 (79)	K	A	N	MEx	UC	–	VAS at rest (0–100), 2	KOOS ADL (0–100), 2	Strength, 2	–
Messier et al. [86]	69 (6)	31 (6)	103 (74)	K	I	N	Ae	Str	–	Pain intensity (1–6), 3	–	–	–
Moghadam and Shojaedin [87]	67 (–)	25 (–)	20 (–)	K	A	N	Ae	UC	–	–	–	6MWT (m), 2	–
Munukka et al. [88]^a^ Waller et al. [89]	64 (–)	27 (–)	87 (100)	K	I	N	Str	UC	–	KOOS pain (0–100), 4	KOOS ADL (0–100), 4	Walking speed (m/s), 4	KOOS QoL (0–100), 4
Oida et al. [90]	74 (5)	25 (4)	88 (86)	K	I	N	MEx	UC	–	–	WOMAC (0–300), 3	Timed walk (s), 3	–
Oosting et al. [91]	76 (6)	28 (–)	30 (80)	H	UClr	Y	MEx	UC	–	NRS (0–10), 1.25	HOOS ADL fx (0–100), 1.25	6MWT (m), 1.25	HOOS QoL (0–100), 1.25
O’Reilly et al. [92]	62 (10)	– (–)	180 (66)	K	UClr	N	Str	UC	–	WOMAC pain (0–20), 6	WOMAC fx (0–68), 6	Quadriceps strength, 6	SF-36 mental health, 6
Petrella and Bartha [93]	74 (5)	– (–)	179 (58)	K	I	N	Fl/Sk	MEx	–	WOMAC pain, 8	WOMAC fx, 8	Timed walk (s), 8	–
Rapp et al. [94]	60 (8)	– (–)	39 (56)	K	A	N	Str	UC	–	VAS (0–10), 2	–	Strength, 2	–
Rathi et al. [95]	54 (7)	– (–)	20 (–)	K	I	N	Ae	MEx	–	NRS, 0.5	WOMAC fx (0–28), 1.5	Strength, 0.5	–
Rogers et al. [96]	71 (11)	33 (7)	20 (80)	K	UClr	N	Fl/Sk	Str	–	WOMAC pain (0–20), 2	WOMAC fx (0–68), 1.5	Timed up and go test (s), 2	–
Rogers et al. [97]	70 (9)	29 (–)	33 (61)	K	A	N	Fl/Sk	MEx	Str	WOMAC pain (0–20), 2	WOMAC fx (0–68), 2	Timed up and go (s), 2	–
Rogind et al. [98]	71 (7)	27 (4)	25 (84)	K	A, I	N	MEx	UC	–	VAS at rest (0–10), 3	Algofunctional index, 3	20-m walk speed (m/s), 3	–
Rooij et al. [99]	64 (–)	36 (–)	126 (75)	K	A	N	MEx	UC	–	NRS (0–10), 2.5	WOMAC fx (0–68), 2.5	6MWT (m), 2.5	–
Rosedale et al. [100]	65 (10)	31 (7)	158 (56)	K	I	Y	MEx	UC	–	KOOS pain (0–100), 3	KOOS fx (0–100), 3	–	KOOS QoL (0–100), 3
Salacinski et al. [101]	58 (10)	24 (–)	37 (73)	K	I	N	Ae	UC	–	VAS at rest (0–100), 3	WOMAC fx (0–100), 3	Walking speed (m/s), 3	KOOS–QoL (0–100), 3
Salli et al. [102] Salli et al. [103]	57 (7)	32 (–)	75 (77)	K	A, I	N	Str	UC	–	VAS at rest, 2	WOMAC fx, 2	Strength (180^o^/s peak torque), 2	SF-36 mental health, 2
Samut et al. [104]	60 (7)	32 (–)	42 (100)	K	A	N	Ae	Str	–	VAS (0–10), 1.5	WOMAC (total) (0–96), 1.5	6MWT (m), 1.5	–
Sayers et al. [105]	67 (7)	31 (7)	45 (56)	K	A	N	Fl/Sk	Str	–	WOMAC pain (0–20), 3	WOMAC fx (0–68), 3	Walking speed (s), 3	–
Schilke et al. [106]	66 (–)	– (–)	23 (74)	K	UClr	N	Str	UC	–	Osteoarthritis screening index pain, 2	Arthritis Impact Measurement Scales activity, 2	Strength, 2	–
Sekir and Gur [107]	60 (9)	– (–)	22 (73)	K	A	N	Fl/Sk	UC	–	VAS (0–100) after inactivity, 1.5	NRS subjective functional rating (0–10), 1.5	Timed walk (s), 1.5	–
Simão et al. [108]	72 (6)	28 (–)	35 (80)	K	A, I	N	Str	UC	–	WOMAC pain (0–500), 3	WOMAC fx (0–1700), 3	6MWT (m), 3	–
Singh et al. [109]^b^ (female, male)	57 (6)	28 (3)	200 (69)	K	UClr	N	Ae	MEx	–	VAS, 2	WOMAC fx, 2	Strength (isotonic), 2	–
Skoffer et al. [110]	70 (–)	31 (–)	59 (61)	K	I	Y	Str	UC	–	KOOS pain (0–100), 1	KOOS ADL (0–100), 1	6MWT (m), 1	KOOS QoL (0–100), 1
Sung-Bum et al. [111]	65 (3)	– (–)	14 (100)	K	A, I	N	Fl/Sk	UC	–	VAS, 2-month	–	Strength (isokinetic), 2	–
Swank et al. [112]	63 (7)	34 (–)	71 (65)	K	UClr	Y	MEx	UC	–	VAS (after walk test) (1–10), 1.5, outlier	–	6MWT (m), 1.5	–
Takacs et al. [113]	67 (–)	29 (–)	40 (80)	K	I	N	Fl/Sk	UC	–	NRS (0–10), 2.5	WOMAC fx (0–68), 2.5	Composite peak lower limb strength, 2.5	–
Teirlinck et al. [114]	66 (–)	28 (–)	203 (59)	H	A	N	MEx	UC	–	NRS (0–10), 1.5	HOOS fx (0–100), 1.5	Timed up and go (s), 12	EQ-5D, 1.5
Teixeira et al. [115]	65 (9)	30 (6)	159 (65)	K	A, I	N	Fl/Sk	MEx	–	–	WOMAC fx—single item, 1.5	–	–
Kuptniratsaikul et al. [116]	68 (6)	– (–)	392 (78)	K	I	N	Str	UC	–	Pain score, 2	Functional incapacity score (0–20), 2	6MWT (m), 2	–
Thorstensson et al. [117]	56 (6)	30 (–)	61 (51)	K	I	N	MEx	UC	–	KOOS pain (0–100), 1.5	KOOS (ADL) (0–100), 1.5	One leg semi squatting, 1.5	SF-36 mental health, 1.5
Topp et al. [118]	63 (11)	– (–)	102 (73)	K	A	N	Str	UC	–	WOMAC pain (0–20), 4	WOMAC fx (0–68), 4	Up stairs (s), 4	–
Topp et al. [119]	64 (7)	32 (6)	54 (69)	K	UClr	Y	MEx	UC	–	VAS (0–10) at 6MWT, 1	–	6MWT (m), 1	–
Tsauo et al. [120]	62 (9)	27 (5)	54 (87)	K	A, I	N	Fl/Sk	MEx	–	WOMAC pain (0–500), 2	WOMAC fx (0–1700), 2	60-m walking (s), 2	–
Vaittianadane et al. [121]	– (–)	– (–)	60 (–)	K	UClr	N	Fl/Sk	MEx	–	–	WOMAC (total), 1.5	Timed up and go (s), 1.5	–
Van Baar et al. [122]^a^ Van Baar et al. [123]	68 (9)	– (–)	201 (78)	K, H	A	N	MEx	UC	–	VAS past week (0–100), 3	Influence of rheumatic disease on general health and lifestyle disability (−28–7), insufficient data	Muscle strength (knee), 3	–
Wallis et al. [124]	68 (–)	34 (–)	46 (44)	K	I	N	Ae	UC	–	NRS (0–10), 3	WOMAC ADL (0–68), 3	40-m walk test (m/s), 3	EQ-5D (0–1), 3
Wang et al. [125]	66 (12)	– (–)	38 (84)	K, H	UClr	N	MEx	UC	–	VAS (0–100), 1.5	Multidimensional health assessment questionnaire, 1.5	Strength knee extension, 1.5	–
Wang et al. [126]	68 (6)	26 (2)	78 (86)	K	UClr	N	MEx	UC	–	KOOS pain (0–100), 1.5	KOOS ADL (0–100), 1.5	6MWT (m), 1.5	KOOS QoL (0–100), 1.5
Weidenhielm et al. [127]	64 (5)	– (–)	39 (51)	K	UClr	Y	MEx	UC	–	Pain 10-grade scale (walking), 3	–	Max walking speed (m/min), 3	–
Weng et al. [128]	64 (8)	– (–)	132 (80)	K	A	N	Fl/Sk	MEx	–	VAS after walking (0–10), 2	Lequesne index (1–26), 2	Peak torque, 2	–
Wortley et al. [129]	69 (6)	32 (6)	31 (71)	K	A, I	N	MB	Str	–	WOMAC pain, 2.5	WOMAC fx, 2.5	6MWT (m), 2.5	–

*A* American College of Rheumatology, *ADL* activity of daily living, *Ae* Aerobic, *AQoL* Assessment of Quality of Life, *BMI* body mass index, *fem* female, *Fl/Sk* flexibility and skills, *fx* function, *H* hip, *HOOS* Hip disability and Osteoarthritis Outcome Score, *I* imaging criteria, *ICOAP* Measure of Intermittent and Constant Osteoarthritis Pain, *Jt* joint, *K* knee, *KOOS* Knee injury and Osteoarthritis Outcome Score, *m* meter, *MB* mind–body, *MCS* mental composite score, *MEx* mixed exercise, *n* sample size, *N* no, *NHP* Nottingham Health Profile questionnaire, *NRS* Numeric rating score, *QoL* quality of life, *s* seconds, *SD* standard deviation, *SF*-*36* 36-item short form survey, *Str* strengthening; *UC* usual care, *UClr* unclear, *VAS* visual analogue scale, *WOMAC* Western Ontario & McMaster Universities Osteoarthritic Index, *Y* yes, *6MWT* 6-minute walk test

^a^Trials with multiple reporting available

^b^Two sets of comparison were obtained

**Table 2:** Characteristics of studies by outcomes

	Pain	Function	Performance	QoL
No. of comparisons	97	97	105	42
Versus usual care	70	67	74	34
Versus another exercise	27	30	31	8
No. of trials (no. participants)	89 (7184)	87 (7153)	95 (6760)	40 (3190)
Knee	75 (5607)	73 (5733)	78 (5156)	30 (2073)
Hip	8 (703)	9 (754)	10 (905)	7 (585)
Both	6 (874)	5 (666)	7 (699)	3 (532)
Age, median (IQR), years	64.9 (62.0–68.7)	64.9 (62.0–69.1)	65.1 (62.4–69.8)	65.3 (62.0–69.7)
BMI, median (IQR), kg/m^2^	29.0 (27.1–31.5)	29.4 (27.2–31.5)	29.0 (27.1–31.5)	29.5 (27.1–31.5)
Female, median (IQR), %	73.0 (61.0–80.7)	73.4 (62.8–81.0)	73.0 (64.1–81.0)	73.7 (61.0–93.2)
Study design				
2 arms	86	83	91	39
3 arms	3	4	4	1

Data presented for each outcome excludes outliers and un-extractable data

*BMI* body mass index, *IQR* interquartile range, *QoL* quality of life

**Fig. 3:**
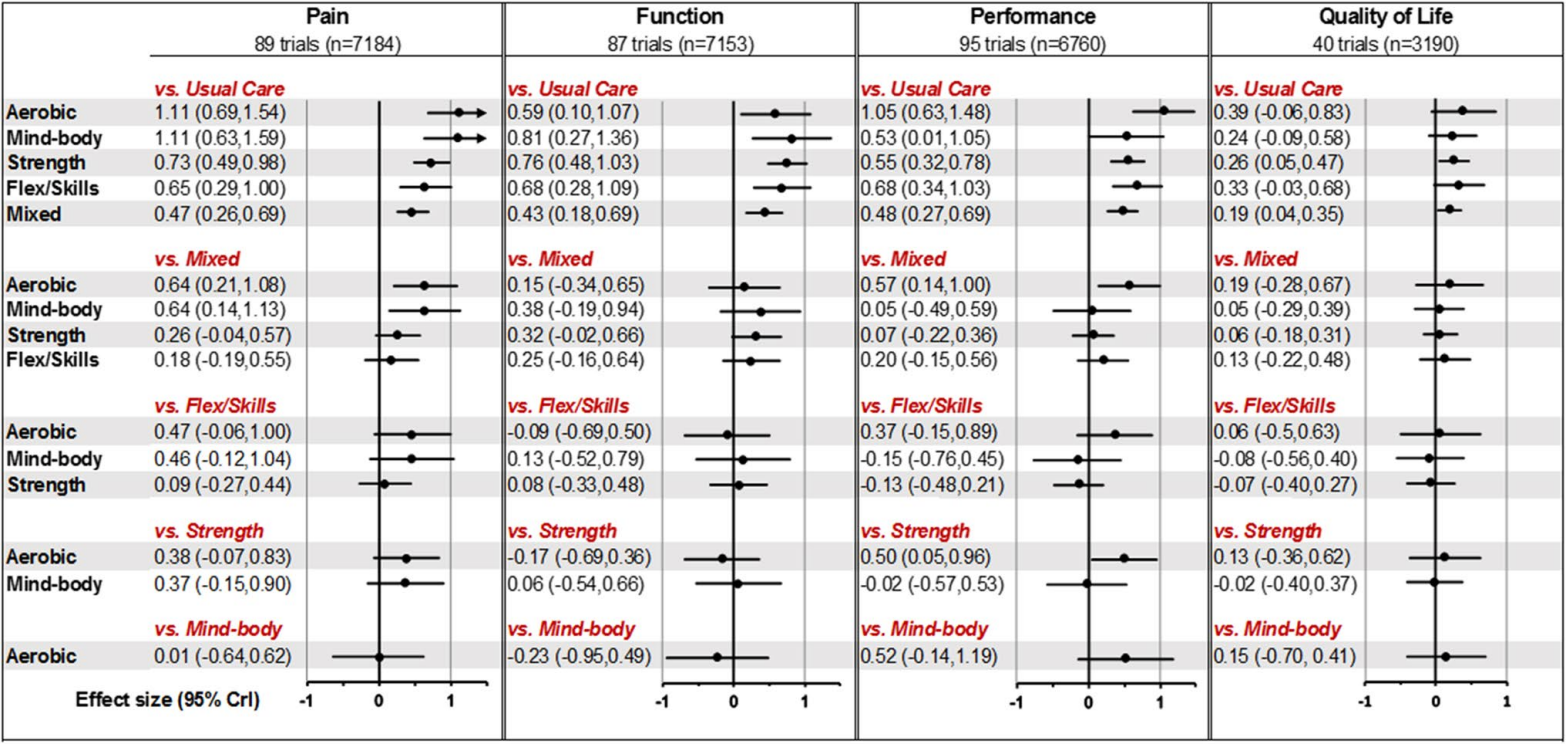
Effect size of different exercise types versus different comparators presented in standardised means difference (95% credibility interval). *Flex/Skills* flexibility and skills exercises, *n* number analysed

The efficacy of different exercises compared with usual care and each other is represented in Fig. 3. For pain, function and performance, all types of exercise were significantly better than usual care, the ES ranging from ES 0.4–1.1. The largest effect was observed for aerobic and mind–body exercise for pain and function. By contrast, the benefits of exercise on QoL were not as marked, with the magnitude of ES ranging from 0.2 to 0.4. Strengthening and flexibility/skill exercises had a moderate ES, whereas mixed exercise gave the minimum ES for all outcomes and was significantly less effective than aerobic or mind–body exercise for pain. The median ranking largely corresponded to the magnitude of ES shown by each exercise. Aerobic was the best-ranked exercise for pain and performance, whereas mind–body was also the best-ranked for pain and self-reported function. Strengthening and flexibility/skill generally received mid-level rankings while mixed exercise was the lowest ranked exercise, superior only to usual care (ESM Appendix 4). Meta-regression demonstrated significant trend for pain (*p* = 0.01) but not for three other outcomes (function, *p* = 0.07; performance, *p* = 0.06; QoL, *p* = 0.65), according to the effect sizes of outcome in descending order. Evidence of lack of model fit was found for pain ($\bar{D}_{\text{res}}$: 189.3, 185 data points; deviant studies were mainly small studies), performance ($\bar{D}_{\text{res}}$: 201.1, 194 arm-level data points; deviant study recruited younger than average patients—mean age 40 years), and QoL ($\bar{D}_{\text{res}}$: 86.3, 81 data points; possibly due to non-homogeneous groups). The model fit for function, on the other hand, was good ($\bar{D}_{\text{res}}$: 183.2, 182 data points). There was significant heterogeneity for all outcomes with the mean between-studies standard deviation ranging from 0.25 to 0.74. No disagreements were found between direct and indirect evidence (ESM Appendix 5) or between estimates from different study designs.

Physician and participant blinding was not achieved in any study (ESM Appendix 6). The risk of bias assessment for individual items per article is detailed in ESM Appendix 7. Sample size, allocation concealment and SD imputation were used for assessing the robustness of the NMA estimate. As there were only seven studies with sample size > 100/arm, we undertook a sensitivity analysis based on ≥ 30 participants/arm—a consensus of the minimum sample size for a trial [131]. The analysis as summarised in ESM Appendix 8 suggested that the results obtained are robust.

Subgroup analysis by joint confirmed the exercise benefits in knee OA for pain, self-reported function and performance, whereas substantial uncertainty for benefits was observed in hip OA. In addition, exercise appeared to be more beneficial among participants who were not awaiting TJR compared with those who were (Table 3).

**Table 3:** Subgroup analysis by joint and recruitment

	Effect size (95% credibility interval)			
	Joint		Recruitment	
	Knee OA	Hip OA	Not awaiting TJR	Awaiting TJR
Pain	75 trials (*n* = 5607)	8 trials (*n* = 703)	75 trials (*n* = 6393)	14 trials (*n* = 791)
Aerobic	1.16 (0.70, 1.61)		1.15 (0.73, 1.59)	
Mind–body	1.30 (0.73, 1.86)		1.13 (0.65, 1.61)	
Strength	0.76 (0.50, 1.02)	0.53 (− 0.74, 1.80)	0.81 (0.54, 1.08)	0.46 (− 0.28, 1.18)
Flex/skills	0.69 (0.31, 1.07)		0.70 (0.33, 1.07)	0.58 (− 1.20, 2.35)
Mixed	0.57 (0.29, 0.85)	0.12 (− 0.36, 0.62)	0.52 (0.29, 0.76)	0.25 (− 0.42, 0.93)
Heterogeneity	0.67 (0.54, 0.82)	0.54 (0.22, 1.20)	0.62 (0.51, 0.77)	0.81 (0.47, 1.37)
Function	73 trials (*n* = 5733)	9 trials (*n* = 754)	76 trials (*n* = 6564)	11 trials (*n* = 589)
Aerobic	0.64 (0.11, 1.17)		0.63 (0.19, 1.07)	0.12 (− 3.11, 3.38)
Mind–body	0.93 (0.27, 1.59)		0.83 (0.35, 1.30)	
Strength	0.78 (0.47, 1.09)	0.69 (− 0.17, 1.54)	0.72 (0.46, 0.99)	0.90 (− 0.58, 2.36)
Flex/skills	0.74 (0.29, 1.19)		0.68 (0.33, 1.03)	
Mixed	0.55 (0.21, 0.89)	0.15 (− 0.17, 0.46)	0.46 (0.23, 0.69)	0.09 (− 1.53, 1.71)
Heterogeneity	0.81 (0.67, 0.98)	0.32 (0.05, 0.75)	0.61 (0.50, 0.74)	1.69 (1.00, 2.97)
Performance	78 trials (*n* = 5208)	10 trials (*n* = 905)	81 trials (*n* = 6331)	14 trials (*n* = 682)
Aerobic	1.12 (0.61, 1.62)	0.81 (0.23, 1.42)	1.05 (0.62, 1.49)	
Mind–body	0.68 (0.03, 1.31)		0.53 (0.01, 1.07)	
Strength	0.60 (0.33, 0.87)	0.29 (− 0.13, 0.75)	0.51 (0.25, 0.77)	0.78 (0.13, 1.43)
Flex/skills	0.76 (0.38, 1.14)		0.66 (0.30, 1.03)	0.90 (− 0.72, 2.53)
Mixed	0.60 (0.31, 0.90)	0.17 (− 0.04, 0.41)	0.50 (0.27, 0.74)	0.35 (− 0.26, 0.97)
Heterogeneity	0.72 (0.58, 0.87)	0.18 (0.01, 0.51)	0.65 (0.53, 0.79)	0.71 (0.41, 1.21)
Quality of life	30 trials (*n* = 2073)	7 trials (*n* = 585)	30 trials (*n* = 2620)	10 trials (*n* = 570)
Aerobic	0.39 (− 0.13, 0.93)		0.38 (− 0.02, 0.79)	
Mind–body	0.37 (− 0.11, 0.86)		0.25 (− 0.05, 0.55)	
Strength	0.27 (0.00, 0.54)	0.30 (− 0.37, 0.97)	0.36 (0.12, 0.62)	0.13 (− 0.40, 0.66)
Flex/skills	0.35 (− 0.10, 0.80)		0.41 (0.07, 0.74)	
Mixed	0.25 (− 0.02, 0.52)	0.06 (− 0.21, 0.36)	0.22 (0.07, 0.38)	0.10 (− 0.56, 0.79)
Heterogeneity	0.35 (0.19, 0.54)	0.19 (0.00, 0.70)	0.19 (0.03, 0.36)	0.53 (0.19, 1.09)

Heterogeneity presented as between-studies standard deviation and 95% credibility interval (CrI)

*Flex/Skills* flexibility/skills exercise, *n* number of participants analysed, *OA* osteoarthritis, *TJR* total joint replacement

## Discussion

This NMA confirms that exercise is beneficial for people with knee and hip OA for outcomes of pain, function, performance and QoL. In additon, we have found (1) aerobic and mind–body exercise have the largest ES for improvements in pain and function; (2) strengthening and flexibility/skill exercises improve multiple outcomes to a varying degree; and (3) mixed exercise (more than one core type) is the least effective exercise across all outcomes and is significantly inferior to aerobic and mind–body exercise for pain.

The results of this NMA differ from the previous NMA by Uthman et al. [8] for the following possible methodological reasons. Firstly, this NMA was primarily designed to examine the relative efficacy between exercises in knee and hip OA, whereas Uthman et al. set out to examine the conclusiveness of the available evidence for exercise using trial sequential analysis. Secondly, our study included 103 trials, whereas the previous NMA included only 60. Thirdly, we used a different exercise classification. Our classification was based on the ACSM criteria [11] but included an additional mind–body exercise and a ‘mixed’ exercise category (that grouped all exercise combinations together irrespective of whether it was two or more different types of exercise). The previous review, on the other hand, examined only three types of exercise (aerobic, flexibility and strengthening) either individually or in combinations of two, or all three. Their results showed that combinations of any two types of exercise tended to have smaller ESs and lower probability of being the best, whereas when all three were combined the overall ES was considerably larger. Fourthly, the previous review used non-exercise controls, which could include other interventions (e.g. patient education, electrotherapy), whereas we used usual care with no new interventions (e.g. ‘waiting-list’ or no intervention apart from usual care/activities). Estimation performed in this way is more precise as treatment effects vary with the type of controls, even with inert agents [132]. Finally, we examined four outcomes (pain, self-reported function, observed performance and QoL), whereas the previous review examined only two (pain and function). Both reviews agree that the effect of exercise depends on the types of exercise or components of the exercise programme. Our results align with other conventional systematic reviews and meta-analyses where aerobic [133] and mind–body exercise [134] tend to have larger effect sizes than strengthening exercise, and mixed exercise tends to have the lowest effect size for pain [5]. Also in line with the literature is the smaller effect size and greater uncertainties of exercise benefits in hip compared with knee OA [4, 135], which still requires further investigation.

A novel finding from this NMA is that we were able to demonstrate that mind–body exercise had similar effects to aerobic exercise for pain. Mind–body exercise such as tai chi and yoga can be characterised as low to moderate intensity exercise performed with an intentional awareness (mindfulness) on breathing and slow controlled movement [136]. Although the underlying mechanism remains unclear, the effect of both aerobic and mind–body exercise may be attributable to the potential of these exercises to influence altered central elements such as central pain sensitisation, sleep disturbance, and mood disorders [137, 138]. Pain experience as well as level of function and QoL are the results of interactions between these central impairments and peripheral pain mechanisms [139, 140]. As aerobic and mind–body exercise could influence both central and peripheral pain mechanisms, this additive effect may explain their additional benefits over other exercises that predominantly address only joint level deficits.

There is no satisfactory biological explanation for the poor efficacy of mixed exercise across all outcomes, particularly when considering that there are many domains of physical impairment in people with OA. However, it may be that the lack of response to mixed exercise reflects flawed implementation of the programme, such that intensity of the individual components was insufficient or poorly adhered to due to the complexity of the regimen compared with a single exercise programme.

There are limitations to this NMA. A key limitation is that we were fully reliant on author descriptions for the classification of exercises and control groups. Exercise programmes and ‘usual care’ are not standardised and vary considerably between studies. Even when the focus of exercise is strength improvement, it is typical to also find some elements of flexibility and/or aerobic exercise included in the programme. As far as possible, we adhered to the classification presented by the authors. The decision to group different types of controls, such as waiting list, usual physical activity and usual care, together for the analysis is open to question. Unlike non-pharmacological treatments for mental health, where a difference between non-treatment and waiting-list controls has been observed [141], no such distinction has been reported for exercise interventions in OA. Instead, many published reports in OA extend controls to include other types of non-exercise interventions (e.g. patient education and behavioural therapy) rather than limiting them to ‘usual care’ [4, 142]. Secondly, the estimates for aerobic, mind–body and flexibility/skill exercises were open to considerable uncertainty with wide credibility intervals as the number of studies were small. However, examination of exercise rankings using different approaches (i.e. probability of the exercise being the best, highest median ranking, or magnitude of ES) showed that the estimates were generally in agreement, supporting the trend observed. Another caveat is that we did not fully explore the reasons for heterogeneity because efforts to identify covariates for exercise effect in OA have generally been unsuccessful in many meta-analyses [8, 143]. This probably requires more sophisticated analytical approaches and warrants separate reporting. Finally, the focus of the included studies was relatively short term and involved mainly single-joint OA. Therefore, we could not determine whether the observed differences between exercises would persist in the longer term or whether people with knee plus hip OA would attain similar exercise benefits.

## Conclusions

In conclusion, this NMA confirms that exercise therapy has clear benefits for people with knee and hip OA and also shows that the magnitude of effect varies according to type of exercise and outcome of interest. Aerobic and mind–body exercises were found to be the best for pain and function, whereas strengthening and flexibility/skill exercises are potentially next best for multiple outcomes. Mixed exercise is the least effective exercise for knee and hip OA but is still superior to usual care for all outcomes and therefore remains an acceptable option for patients who do not respond well to single-component exercises. The findings of this review may help clinicians guide their prescription of exercise type with respect to treatment outcomes. Further research is warranted to confirm if the hierarchy observed are consistent across all patients with OA.

## Electronic supplementary material

Below is the link to the electronic supplementary material.

Supplementary material 1 (PDF 543 kb)
