# Supplementary material for: Relative Efficacy of Different Exercises for Pain, Function, Performance and Quality of Life in Knee and Hip Osteoarthritis: Systematic Review and Network Meta-Analysis
Source: Sports Med. 2019 Mar 4;49(5):743–61. doi: 10.1007/s40279-019-01082-0 (PMC6459784; doi:10.1007/s40279-019-01082-0)
Supplement: Supplementary file 1 — Supplementary material 1 (PDF 543 kb) [file 40279_2019_1082_MOESM1_ESM.pdf]

Article title:

Relative efficacy of different exercises for pain, function, performance and quality of life in knee and hip osteoarthritis: Systematic review and Network meta-analysis

Journal name:

Sports Medicine

Authors:

Siew-Li Goh, Monica SM Persson, Joanne Stocks, Yunfei Hou, Nicky J Welton, Jianhao Lin, Michelle C Hall, Michael Doherty, Weiya Zhang

Affiliation and email address of corresponding author:

Prof Weiya Zhang,

Arthritis Research UK Pain Centre, Academic Rheumatology,

Email: Weiya.Zhang@nottingham.ac.uk

Appendix 1: Medline Search Strategy

1. exercise/ or physical conditioning, human/
2. Circuit-Based Exercise/
3. exercise therapy/ or motion therapy, continuous passive/ or muscle stretching exercises/ or plyometric exercise/ or resistance training/ or hydrotherapy/ or rehabilitation/ or "activities of daily living"/ or dance therapy/
4. muscle strength/ or physical endurance/ or anaerobic threshold/ or exercise tolerance/ or physical fitness/ or postural balance/ or posture/ or psychomotor performance/ or "range of motion, articular"/
5. Pliability/
6. movement/ or motor activity/ or exercise/
7. Physical Exertion/
8. Mind-Body Therapies/
9. running/ or jogging/ or swimming/ or walking/
10. Isometric Contraction/
11. exercise movement techniques/ or breathing exercises/ or qigong/ or tai ji/ or yoga/ or pilates
12. propriocepti$.ab,ti.
13. balanc$.ab,ti.
14. aqua$.ab,ti.
15. cycl$.ab,ti.
16. aerobic.ab,ti.
17. strength$.ab,ti.
18. (tai-ji or taiji or taijiquan or tai ji quan or tai chi or taichi or t ai chi or t'ai chi or tai chi chuan).ab,ti.
19. (qigong or qi gong or chi kung or chikung or ch i kung or ch'i kung).ab,ti.
20. therap$.ab,ti.
21. physiotherap$.ab,ti.
22. train$.ab,ti.
23. neuromuscular training.ab,ti.
24. treadmill.ab,ti.
25. 1 or 2 or 3 or 4 or 5 or 6 or 7 or 8 or 9 or 10 or 11 or 12 or 13 or 14 or 15 or 16 or 17 or 18 or 19 or 20 or 21 or 22 or 23 or 24
26. Osteoarthritis, Hip/ or Osteoarthritis, Knee/
27. coxarthritis.mp.
28. coxarthr$.ab,ti.
29. gonarthr$.ab,ti.
30. (knee$ adj3 pain).ab,ti.
31. (hip adj3 pain).ab,ti.
32. osteoarthr$.ab,ti.
33. degenerative joint disease?.ab,ti.
34. osteoarthritis/
35. (Osteoarthriti$ or OA or osteo arthriti$ or osteoarthros$ or osteo arthros$ or arthropath$ or arthrosis or arthroses).ti,ab.
36. Hip Joint/
37. Hip/
38. hip$.ab,ti.
39. Knee/

40. knee$.ab,ti.
41. knee joint/ or patellofemoral joint/
42. 26 or 27 or 28 or 29 or 30 or 31
43. 32 or 33 or 34 or 35
44. 36 or 37 or 38 or 39 or 40 or 41
45. 43 and 44
46. 42 or 45
47. randomized controlled trial.pt.
48. controlled clinical trial.pt.
49. (placebo or (standard adj3 care)).ab.
50. clinical trials as topic.sh.
51. random$.ab,ti.
52. trial$.ab,ti.
53. RCT.ab,ti.
54. 47 or 48 or 49 or 50 or 51 or 52 or 53
55. limit 54 to humans
56. 25 and 46 and 55

Appendix 2: Bayesian codes

a. **Arm-based data**

```
#Random effect
#multi-arm (include 3 and 4 arms)
# Random effects model for multi-arm trials, continuous outcome
# SMD

model{
for(i in 1:ns){
   w[i,1] <- 0
   delta[i,1] <- 0
   mu[i] ~ dnorm(0,.0001)   # vague priors for 24 trial baselines
   for (k in 1:na[i])  {
     var[i,k] <- pow(se[i,k],2)
     prec[i,k] <- 1/var[i,k]         # set precisions
            y[i,k] ~ dnorm(phi[i,k], prec[i,k]) # normal likelihood
            phi[i,k] <- theta[i,k] * Pooled.sd[i]
            theta[i,k] <- mu[i] + delta[i,k]
# deviance arm k, study i
     dev[i,k] <- (y[i,k]-phi[i,k])*(y[i,k]-phi[i,k])/var[i,k]
   }
  for (k in 2:na[i]){
# distributions for trial-specific SMD
    delta[i,k] ~ dnorm(md[i,k], taud[i,k])
    md[i,k] <- (d[t[i,k]] - d[t[i,1]]) + sw[i,k]
#precision of diff in means distributions
   taud[i,k] <- tau *2*(k-1)/k
#adjustment, multi-arm RCTs
   w[i,k] <- delta[i,k] - d[t[i,k]] + d[t[i,1]]
# cumulative adjustment for multi-arm trials
   sw[i,k] <-sum(w[i,1:k-1])/(k-1)
  }
  resdev[i] <- sum(dev[i, 1:na[i]])     # residual deviance for study i
}
d[1]<-0

for (k in 2:nt){d[k] ~ dnorm(0,.0001) } # vague priors for basic parameters
sd.d ~ dunif(0,10)                      # vague prior for RE st dev
var.d <- pow(sd.d,2)
tau <- 1/var.d


# overall residual deviance
totresdev <- sum(resdev[])
# all pairwise differences
for (c in 1:(nt-1)) { for (k in (c+1):nt) {pwdiff[c,k] <- (d[k]-d[c])  } }


for (k in 1:nt) {
```

```
 # assumes events are “good”
rk[k] <- nt+1-rank(d[],k)
best[k] <- equals(rk[k],1) #calculate probability that treat k is best
}
}
```

b. Arm-based and trial-based data

```
#Random effect
#multi-arm (include 3 and 4 arms)
# Random effects model for multi-arm trials, continuous outcome
# SMD

model{
for(i in 1:ns.a){   #ARM-LEVEL DATA
   w[i,1] <- 0
   delta[i,1] <- 0
   mu[i] ~ dnorm(0,.0001)   # vague priors for trial baselines
   for (k in 1:na[i])  {
     var[i,k] <- pow(se[i,k],2)
     prec[i,k] <- 1/var[i,k]        # set precisions
             y[i,k] ~ dnorm(phi[i,k], prec[i,k]) # normal likelihood
             phi[i,k] <- theta[i,k] * Pooled.sd[i]
             theta[i,k] <- mu[i] + delta[i,k]
# deviance arm k, study i
     dev[i,k] <- (y[i,k]-phi[i,k])*(y[i,k]-phi[i,k])/var[i,k]
   }
  for (k in 2:na[i]){
# distributions for trial-specific SMD
    delta[i,k] ~ dnorm(md[i,k], taud[i,k])
    md[i,k] <- (d[t[i,k]] - d[t[i,1]]) + sw[i,k]
#precision of diff in means distributions
   taud[i,k] <- tau *2*(k-1)/k
#adjustment, multi-arm RCTs
   w[i,k] <- delta[i,k] - d[t[i,k]] + d[t[i,1]]
# cumulative adjustment for multi-arm trials
   sw[i,k] <-sum(w[i,1:k-1])/(k-1)
  }
  resdev.a[i] <- sum(dev[i, 1:na[i]])     # residual deviance for study i
}


for(i in 1:ns.t2){   #TRIAL-LEVEL DATA, 2-arm trials
             y.t[i,2] ~ dnorm(phi.t[i,2], prec.t[i,2]) # normal likelihood
# deviance arm k, study i
     resdev.t[i] <- (y.t[i,2]-phi.t[i,2])*(y.t[i,2]-phi.t[i,2])*prec.t[i,2]
```

```
  }

for(i in (ns.t2+1):(ns.t2+ns.t3)) {    #TRIAL-LEVEL DATA: 3-ARM TRIALS
    for (k in 1:(na.t[i]-1)) {    # set variance-covariance matrix
        for (j in 1:(na.t[i]-1)) {
            Sigma[i,j,k] <- V[i]*(1-equals(j,k)) + var.t[i,(k+1)]*equals(j,k)
          }
      }
    Omega[i,1:(na.t[i]-1),1:(na.t[i]-1)] <- inverse(Sigma[i,,])  #Precision
# multivariate normal likelihood for 3-arm trials
    y.t[i,2:na.t[i]] ~ dmnorm(phi.t[i,2:na.t[i]],Omega[i,1:(na.t[i]-1),1:(na.t[i]-1)])

#Deviance contribution for trial i
    for (k in 1:(na.t[i]-1)){  # multiply vector & matrix
        ydiff[i,k]<- y.t[i,(k+1)] - phi.t[i,(k+1)]
        z[i,k]<- inprod2(Omega[i,k,1:(na.t[i]-1)], ydiff[i,1:(na.t[i]-1)])
                       }
    resdev.t[i]<- inprod2(ydiff[i,1:(na.t[i]-1)], z[i,1:(na.t[i]-1)])
  }

for(i in 1:(ns.t2+ns.t3)){   #TRIAL LEVEL DATA: RANDOM EFFECTS MODEL
   w.t[i,1] <- 0
   for (k in 2:na.t[i])  {
     var.t[i,k] <- pow(se.t[i,k],2)
     prec.t[i,k] <- 1/var.t[i,k]         # set precisions
             phi.t[i,k] <- delta.t[i,k] * Pooled.sd.t[i]
# distributions for trial-specific SMD
    delta.t[i,k] ~ dnorm(md.t[i,k], taud.t[i,k])
    md.t[i,k] <- (d[t.t[i,k]] - d[t.t[i,1]]) + sw.t[i,k]
#precision of diff in means distributions
   taud.t[i,k] <- tau *2*(k-1)/k
#adjustment, multi-arm RCTs
   w.t[i,k] <- delta.t[i,k] - d[t.t[i,k]] + d[t.t[i,1]]
# cumulative adjustment for multi-arm trials
   sw.t[i,k] <-sum(w.t[i,1:k-1])/(k-1)
  }
}

d[1]<-0
for (k in 2:nt){d[k] ~ dnorm(0,.0001) } # vague priors for basic parameters
sd.d ~ dunif(0,10)                         # vague prior for RE st dev
var.d <- pow(sd.d,2)
tau <- 1/var.d
# overall residual deviance
totresdev.a <- sum(resdev.a[])
totresdev.t<-sum(resdev.t[])

# all pairwise differences
for (c in 1:(nt-1)) { for (k in (c+1):nt) {pwdiff[c,k] <- (d[k]-d[c])  } }
for (k in 1:nt) {
```

```
 # assumes events are "good"
rk[k] <- nt+1-rank(d[],k)
best[k] <- equals(rk[k],1) #calculate probability that treat k is best
}

for (c in 1:(nt-1)) {                                    #all possible pair-wise comparison
 for (k in (c+1):nt) {
  SMD[c,k] <- (d[k]-d[c])
                    }
          }
}
```

Appendix 3: Funnel plot for pain, function, performance and quality of life

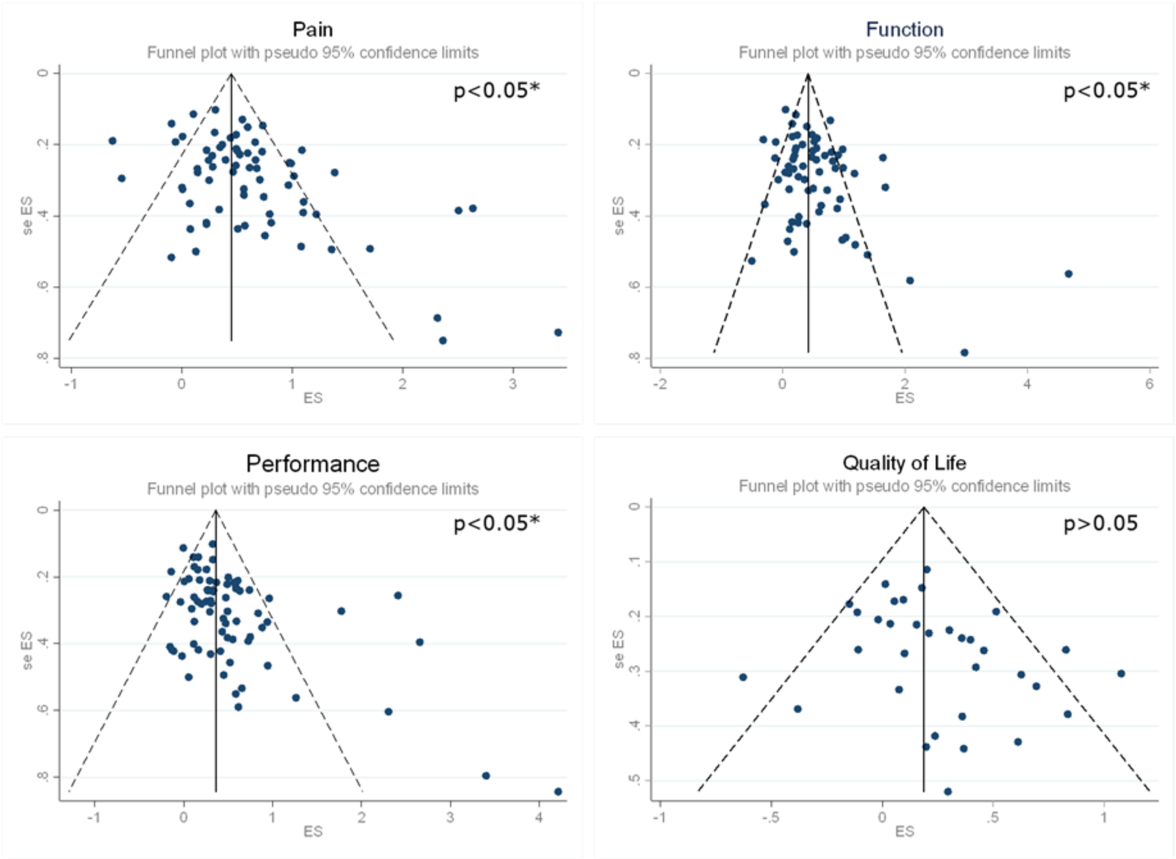

Plot to assess the bias/symmetrical distribution in the evidence of direct comparison between different exercises and usual care.

* Egger’s test suggestive of publication bias when $p<0.05$.

Appendix 4: Median ranking (95% credibility interval) of each exercise for pain, function, performance and quality of life outcomes

	Pain	Function	Performance	Quality of Life
**Aerobic**	1 (1, 3)	4 (1, 5)	1 (1, 3)	2 (1, 6)
**Mind-body**	2 (1, 4)	2 (1, 5)	4 (1, 5)	3 (1, 6)
**Strength**	3 (2, 5)	2 (1, 4)	4 (2, 5)	3 (1, 5)
**Flex/Skills**	4 (2, 5)	3 (1, 5)	2 (1, 5)	2 (1, 6)
**Mixed**	5 (3, 5)	4 (3, 5)	4 (2, 5)	4 (2, 5)
**Usual care**	6 (6, 6)	6 (6, 6)	6 (6, 6)	6 (5, 6)

1= First; 2= Second; 3= Third; 4= Fourth, 5= Fifth, 6= Sixth.
The highest ranking is the best (i.e. if there is no median ranking of 1st, the 2nd ranking will be the best)

Appendix 5: Estimates from direct and indirect comparison for each exercise types relative to usual care control

	Effect size (95% confidence interval)	
	Direct	Indirect
**Pain**		
Aerobic	-0.82 (-1.42, -0.21)	-1.15 (-1.62, -0.68)
Flex/skills	-0.81 (-1.39, -0.23)	-0.47 (-0.83, -0.10)
Mind-body	-0.81 (-1.35, -0.27)	-1.47 (-2.12, -0.81)
Mixed	-0.50 (-0.70, -0.29)	-0.15 (-0.57, 0.28)
Strength	-0.60 (-0.84, -0.36)	-0.84 (-1.28, -0.39)
**Function**		
Aerobic	-0.50 (-1.10, 0.11)	-0.60 (-1.16, -0.04)
Flex/skills	-0.85 (-1.17, -0.07)	-0.57 (-0.95, -0.19)
Mind-body	-0.67 (-1.27, -0.06)	-0.96 (-1.69, -0.22)
Mixed	-0.35 (-0.59, -0.11)	-0.49 (-0.94, -0.05)
Strength	-0.74 (-1.01, -0.47)	-0.50 (-0.99, -0.01)
**Performance**		
Aerobic	-0.86 (-1.47, -0.26)	-0.94 (-1.36, -0.52)
Flex/skills	-0.59 (-1.12, -0.05)	-0.63 (-0.95, -0.31)
Mind-body	-0.54 (-1.05, -0.03)	-0.36 (-1.11, 0.39)
Mixed	-0.40 (-0.60, -0.21)	-0.50 (-0.87, -0.14)
Strength	-0.52 (-0.73, -0.30)	-0.43 (-0.81, -0.05)
**Quality of Life**		
Aerobic	-0.18 (-0.32,-0.04)	-
Flex/skills	-	-0.31 (-0.63, 0.00)
Mind-body	-0.22 (-0.59, 0.15)	-0.18 (-0.77, 0.41)
Mixed	-0.18 (-0.34, -0.03)	-0.19 (-0.61, 0.23)
Strength	-0.23 (-0.43, -0.02)	-0.32 (-0.79, 0.16)

Overlapping of 95% confidence internal indicates no disagreement between direct and indirect evidence

Appendix 6: Risk of bias

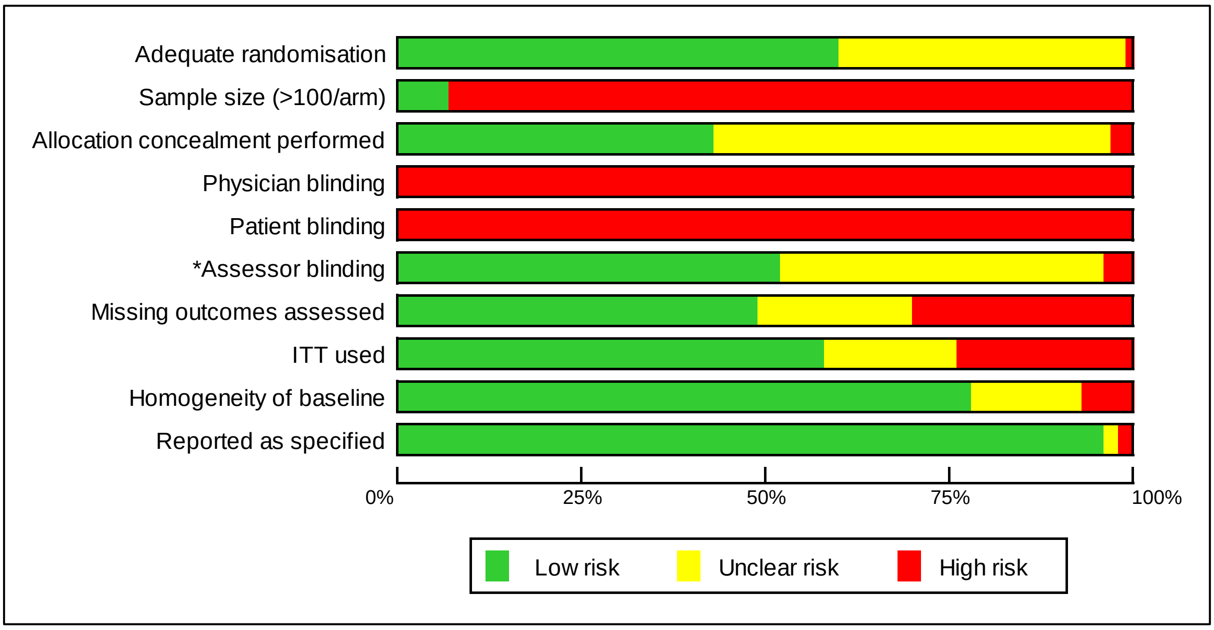

* Data is for studies with objective performance test since assessor blinding is not possible for self-reported outcomes
ITT= intention to treat analysis

Appendix 7: Risk of bias assessment per article and country of publication

Study	Adequate randomization	>100 per group	Concealed Allocation	Physician Blind	Patient Blind	Assessor Blind	Missing Outcome Assess	Intention-to-treat Use	Homogenous Group	Reported as Pre-specified	Country
Abbott 2013 (a)	Yes	No	Unclear	No	No	Yes	Yes	Yes	Yes	Yes	New Zealand
Aglamis 2008	Yes	No	Unclear	Unclear	No	Yes	No	No	Unclear	Yes	Turkey
An 2008	Unclear	No	Unclear	Unclear	No	No	Yes	No	Yes	Yes	China
Aoki 2009	Unclear	No	Unclear	Unclear	No	Yes	Yes	Yes	Yes	Yes	Japan
Arnold 2010	Yes	No	Yes	No	No	Yes	Yes	Yes	Yes	Yes	Canada
Beaupre 2004	Unclear	No	Yes	No	No	Yes	Yes	Yes	Yes	Yes	Canada
Bennell 2010	Yes	No	Yes	No	No	Yes	Yes	Yes	Yes	Yes	Australia
Bennell 2014	Yes	No	Yes	No	No	Yes	Yes	Yes	Yes	Yes	Australia
Bieler 2017	Yes	No	Unclear	No	No	Yes	Yes	Yes	Yes	Yes	Denmark
Börjesson 1996	Yes	No	Unclear	No	No	Unclear	Yes	Yes	Yes	Unclear	Sweden
Braghin 2017	Yes	No	Yes	No	No	Unclear	Unclear	Unclear	Unclear	Yes	Brazil
Bruce-Brand 2012	Yes	No	Unclear	No	No	Yes	Unclear	Unclear	Yes	No	United Kingdom
Calatayud 2017	Yes	No	Unclear	No	No	Unclear	Yes	Yes	Yes	Yes	Denmark
Chaipinyo 2009	Unclear	No	Yes	No	No	Yes	No	Unclear	Unclear	Yes	Thailand
Chen 2014	Yes	No	Yes	No	No	Unclear	Unclear	Yes	Unclear	Yes	Taiwan
Cheung 2016	Yes	No	Yes	No	No	Unclear	Yes	Yes	No	Yes	United States
Chopp-Hurley 2017	Yes	No	Unclear	No	No	Yes	No	Unclear	Yes	Yes	Canada
Christensen 2015	Yes	No	Yes	No	No	Yes	Yes	Yes	Yes	Yes	Denmark
Cochrane 2005	Yes	Yes	Yes	No	No	Yes	Yes	Yes	Yes	Yes	United Kingdom
Corjena 2014	Yes	No	Unclear	No	No	Yes	Yes	Yes	Yes	Yes	United States
D'Lima 1996	Yes	No	Unclear	Unclear	No	Unclear	Yes	Unclear	No	Yes	United States
Duracoglu 2005	Unclear	No	Unclear	No	No	Yes	No	No	Yes	Yes	Turkey
Ebnezar 2012	Yes	Yes	Yes	No	No	Yes	No	No	Yes	Yes	India
Espejo Antunez 2012	Yes	No	No	No	No	Yes	Yes	Yes	Unclear	Yes	Spain
Ettinger 1997	Yes	Yes	Unclear	No	No	Yes	Yes	Yes	Yes	Yes	United States
Evcik 2002	Unclear	No	Unclear	No	No	No	No	No	Yes	Yes	Turkey
Evgeniadis 2008	Yes	No	Yes	No	No	Unclear	No	Yes	Yes	Yes	Greece
Ferrara 2008	Yes	No	Unclear	Unclear	No	Yes	Unclear	Yes	Yes	No	Italy
Fitzgerald 2011	Yes	No	Yes	No	No	Yes	Yes	Yes	Yes	Yes	United States
Fransen 2001	Yes	No	Yes	No	No	Yes	Yes	Yes	Yes	Yes	Australia

Study	Adequate randomization	>100 per group	Concealed Allocation	Physician Blind	Patient Blind	Assessor Blind	Missing Outcome Assess	Intention-to-treat Use	Homogenous Group	Reported as Pre-specified	Country
Fransen 2007	Yes	No	Unclear	No	No	Yes	Yes	Yes	Yes	Yes	Australia
French 2013	Yes	No	Yes	No	No	Yes	Yes	Yes	Unclear	Yes	Ireland
Ghroubi 2008 (a)	Unclear	No	Unclear	No	No	Unclear	No	No	Yes	Yes	Tunisia
Gomiero 2017	Yes	No	Yes	No	No	Unclear	Yes	Yes	Yes	Yes	Brazil
Gondhalekar 2013	Unclear	No	Unclear	No	No	Unclear	No	No	Unclear	Yes	India
Gur 2002	Unclear	No	Unclear	No	No	Unclear	Yes	Yes	Unclear	Yes	Turkey
Hasegawa 2010	Unclear	No	Unclear	No	No	Unclear	Yes	Yes	Yes	Yes	Japan
Henriksen 2014	Yes	No	Yes	No	No	Yes	No	No	No	Yes	Denmark
Hermann 2016	Yes	No	Yes	No	No	Unclear	Yes	Yes	Yes	Yes	Denmark
Hinman 2007	Yes	No	Yes	No	No	Yes	Yes	Yes	Yes	Yes	Australia
Hiyama 2012	Yes	No	Yes	No	No	Yes	Yes	Yes	Yes	Yes	Japan
Hoogeboom 2010	Unclear	No	Yes	No	No	Yes	Yes	Yes	Yes	Yes	Netherland
Huang 2003	Unclear	No	Yes	No	No	Unclear	No	No	Unclear	Yes	Taiwan
Huang 2005	Unclear	No	Yes	No	No	Unclear	No	No	Unclear	Yes	Taiwan
Huang 2017	Yes	Yes	No	Unclear	No	No	Unclear	Unclear	Yes	Yes	China
Hunt 2013	Yes	No	Yes	No	No	Yes	No	Unclear	Yes	Yes	Canada
Ikuta 2015	Unclear	No	Unclear	No	No	Unclear	Unclear	No	Yes	Yes	Japan
Jan 2008	Yes	No	Unclear	No	No	Yes	No	Yes	Yes	Yes	Taiwan
Jan 2009	Yes	No	Unclear	No	No	Yes	Unclear	Yes	Yes	Yes	Taiwan
Jorge 2015	Yes	No	Yes	No	No	Yes	Yes	Yes	Yes	Yes	Brazil
Juhakoski 2011	Yes	No	Yes	No	No	Yes	Yes	Yes	Yes	Yes	Finland
Koli 2015	Yes	No	Unclear	No	No	Yes	No	No	Yes	Yes	Finland
Krasilshchikov 2011	Unclear	No	Unclear	No	No	Yes	Unclear	Unclear	Yes	Yes	Malaysia
Krauss 2014	Unclear	No	Yes	No	No	No	Yes	Yes	Yes	Yes	Germany
Kreindler 1989	No	No	No	No	No	Unclear	Yes	Yes	Yes	Yes	United States
Kumar 2013	Yes	No	Unclear	No	No	No	Yes	Yes	Yes	Yes	India
Lee 2008	Unclear	No	Unclear	No	No	Unclear	No	No	No	Yes	Korea
Lee 2009	Yes	No	Yes	No	No	Yes	Yes	Yes	Yes	Yes	Korea
Lim 2008 (neutral)	Yes	No	Unclear	No	No	Yes	Yes	Yes	Yes	Yes	Australia
Lin 2007	Unclear	No	Unclear	No	No	Unclear	No	No	Yes	Yes	Taiwan
Lin 2009	Yes	No	Yes	No	No	Yes	Yes	Yes	Yes	Yes	Taiwan

Study	Adequate randomization	>100 per group	Concealed Allocation	Physician Blind	Patient Blind	Assessor Blind	Missing Outcome Assess	Intention-to-treat Use	Homogenous Group	Reported as Pre-specified	Country
Lund 2008	Unclear	No	Yes	No	No	Yes	Yes	Yes	Yes	Yes	Denmark
Messier 1997	Unclear	No	Unclear	No	No	Unclear	Unclear	Unclear	Yes	Yes	United States
Moghadam 2017	Unclear	No	Unclear	No	No	Unclear	No	Unclear	Yes	Unclear	Azerbaijan
Munukka 2016	Yes	No	Unclear	No	No	Yes	Yes	Yes	Yes	Yes	Finland
Oida 2008	Yes	No	Unclear	No	No	Unclear	Unclear	No	No	Yes	Japan
Oosting 2012	Unclear	No	Yes	No	No	Yes	No	Yes	Yes	Yes	Netherlands
O'Reilly 1999	Yes	No	Yes	No	No	Unclear	Unclear	Yes	Yes	Yes	United Kingdom
Petrella 2000	Yes	No	Unclear	No	No	Yes	No	Yes	Yes	Yes	United States
Rapp 2009	Unclear	No	Unclear	No	No	Unclear	Yes	Yes	Unclear	Yes	Germany
Rathi 2014	Unclear	No	Unclear	No	No	Unclear	Yes	Yes	Yes	Yes	India
Rogers 2011	Yes	No	Unclear	No	No	Unclear	No	No	Yes	Yes	United States
Rogers 2012	Yes	No	Unclear	No	No	Unclear	Unclear	No	Yes	Yes	United States
Rogind 1998	Yes	No	Unclear	No	No	Yes	Unclear	Yes	Yes	Unclear	Denmark
Rooij 2016	Yes	No	Yes	No	No	Yes	Yes	Yes	Yes	Yes	Netherlands
Rosedale 2014	Yes	No	Yes	No	No	No	Yes	Yes	Yes	Yes	Canada
Salacinski 2012	Yes	No	Unclear	No	No	Unclear	No	No	No	Yes	United States
Salli 2010	Unclear	No	Yes	No	No	Unclear	No	No	Yes	Yes	Turkey
Samut 2015	Unclear	No	Unclear	No	No	Unclear	No	Yes	Yes	Yes	Turkey
Sayers 2012	Yes	No	Unclear	No	No	Yes	Unclear	Yes	Yes	Yes	United States
Schilke 1996	Unclear	No	Unclear	No	No	Unclear	No	No	Unclear	Yes	United States
Sekir 2005	Unclear	No	Unclear	No	No	Unclear	Yes	Yes	Unclear	Yes	Turkey
Simão 2012	Unclear	No	Yes	No	No	Yes	No	Yes	No	Yes	Brazil
Singh 2011 (female)	Unclear	No	Unclear	No	No	Unclear	Unclear	Unclear	Unclear	Yes	Punjab
Skoffer 2016	Yes	No	Yes	No	No	Yes	Yes	Yes	Yes	Yes	Denmark
Sung-Bum 2015	Unclear	No	Unclear	No	No	Unclear	Unclear	Unclear	Yes	Yes	Korea
Swank 2011	Unclear	No	Unclear	No	No	Unclear	Yes	Yes	Yes	Yes	United States
Takacs 2017	Yes	No	Yes	No	No	Yes	Yes	No	Yes	Yes	Canada
Teirlinck 2016	Yes	Yes	Yes	No	No	No	Yes	Yes	Yes	Yes	Netherlands
Teixeira 2011	Unclear	No	Yes	No	No	No	No	No	Yes	Yes	United States
Thamalikitkul 2002	Unclear	Yes	Unclear	No	No	Unclear	Unclear	Unclear	Yes	Yes	Thailand
Thorstensson 2005	Yes	No	Yes	No	No	Unclear	Unclear	Unclear	Yes	Yes	Sweden

Study	Adequate randomization	>100 per group	Concealed Allocation	Physician Blind	Patient Blind	Assessor Blind	Missing Outcome Assess	Intention-to-treat Use	Homogenous Group	Reported as Pre-specified	Country
Topp 2002	Unclear	No	Unclear	No	No	Unclear	Unclear	Unclear	Yes	Yes	United States
Topp 2009	Unclear	No	Unclear	No	No	Unclear	Unclear	Unclear	Yes	Yes	United States
Tsauo 2008	Yes	No	Unclear	No	No	Unclear	No	No	Yes	Yes	Taiwan
Vaittianadane 2014	Unclear	No	Unclear	No	No	Unclear	Unclear	Unclear	Unclear	Yes	India
Van Baar 1998	Yes	Yes	Yes	No	No	Yes	Yes	Yes	Yes	Yes	Netherland
Wallis 2017	Yes	No	Yes	No	No	Yes	Yes	Yes	Yes	Yes	Australia
Wang 2007	Unclear	No	Unclear	No	No	Unclear	No	Yes	Yes	Yes	Taiwan
Wang 2011	Yes	No	Unclear	No	No	Yes	No	No	Yes	Yes	Taiwan
Weidenhielm 1993	Yes	No	Unclear	No	No	Unclear	Yes	Unclear	Yes	Yes	Sweden
Weng 2009	Yes	No	Yes	No	No	Yes	Unclear	Yes	Yes	Yes	Taiwan
Wortley 2013	Unclear	No	Unclear	No	No	Unclear	No	No	Unclear	Yes	United States

Appendix 8: Sensitivity analysis

	Effect size (95% credible interval) *versus usual care*			
	**Primary analysis**	**Sensitivity analysis**		
		**Allocation concealment - low risk**	**SD not imputed**	**Sample size ≥30/arm**
**Pain**	**89 trials (n=7184)**	**41 trials (n=3975)**	**86 trials (n=6666)**	**37 trials (n=5245)**
Aerobic	1.11 (0.69, 1.54)	0.43 (-0.39, 1.26)	1.12 (0.66, 1.57)	0.95 (0.43, 1.47)
Mind-body	1.11 (0.63, 1.59)	1.60 (0.95, 2.24)	1.10 (0.61, 1.60)	1.21 (0.48, 1.94)
Strength	0.73 (0.49, 0.98)	0.50 (0.19. 0.80)	0.74 (0.48, 1.00)	0.53 (0.24, 0.82)
Flex/Skills	0.65 (0.29, 1.00)	0.29 (-0.15, 0.73)	0.65 (0.29, 1.01)	0.35 (-0.12, 0.83)
Mixed	0.47 (0.26, 0.69)	0.40 (0.16, 0.64)	0.46 (0.24, 0.69)	0.27 (0.01, 0.53)
**Function**	**87 trials (n=7153)**	**42 trials (n=3951)**	**85 trials (n=6843)**	**39 trials (n=5342)**
Aerobic	0.59 (0.10, 1.07)	0.22 (-0.52, 0.95)	0.59 (0.10, 1.08)	0.45 (-0.26, 1.15)
Mind-body	0.81 (0.27, 1.36)	1.04 (0.46, 1.6)	0.81 (0.26, 1.37)	1.03 (0.13, 1.94)
Strength	0.76 (0.48, 1.03)	0.60 (0.34, 0.86)	0.75 (0.46, 1.04)	0.65 (0.31, 1.00)
Flex/Skills	0.68 (0.28, 1.09)	0.25 (-0.10, 0.61)	0.66 (0.24, 1.08)	0.60 (0.08, 1.13)
Mixed	0.43 (0.18, 0.69)	0.30 (0.09, 0.51)	0.44 (0.19, 0.70)	0.37 (0.05, 0.70)
**Performance**	**95 trials (n=6760)**	**41 trials (n=3579)**	**93 trials (n=6955)**	**38 trials (n=4911)**
Aerobic	1.05 (0.63, 1.48)	0.65 (-0.18,1.49)	1.06 (0.63, 1.48)	0.95 (0.42, 1.49)
Mind-body	0.53 (0.01, 1.05)	0.66 (-0.15, 1.47)	0.53 (0.02, 1.05)	0.05 (-0.98, 1.08)
Strength	0.55 (0.32, 0.78)	0.44 (0.14, 0.74)	0.53 (0.30,0.76)	0.47 (0.17, 0.77)
Flex/Skills	0.68 (0.34, 1.03)	0.37 (-0.07, 0.82)	0.64 (0.29, 0.99)	0.47 (-0.01, 0.95)
Mixed	0.48 (0.27, 0.69)	0.36 (0.11, 0.60)	0.50 (0.29, 0.71)	0.42 (0.15, 0.69)
**Quality of life**	**40 trials (n=3190)**	**26 trials (n=2486)**	**NA**	**18 trials (n=2281)**
Aerobic	0.39 (-0.06, 0.83)	0.62 (-0.16, 1.42)	-	0.20 (-0.32, 0.73)
Mind-body	0.24 (-0.09, 0.58)	0.42 (-0.11, 0.96)	-	0.00 (-0.40, 0.41)
Strength	0.26 (0.05, 0.47)	0.20 (-0.05, 0.45)	-	0.11 (-0.09, 0.30)
Flex/Skills	0.33 (-0.03, 0.68)	0.19 (-0.23, 0.56)	-	0.34 (0.04, 0.63)
Mixed	0.19 (0.04, 0.35)	0.12 (-0.06, 0.31)	-	0.14 (0.01, 0.27)

Sensitivity analysis performed to show if some underlying assumptions (i.e. high risk of allocation concealment, imputed standard deviation (SD), sample size <30/arm) would significantly alter the conclusion of primary analysis.
